# Surface heterojunction based on n-type low-dimensional perovskite film for highly efficient perovskite tandem solar cells

**DOI:** 10.1093/nsr/nwae055

**Published:** 2024-02-13

**Authors:** Xianyuan Jiang, Qilin Zhou, Yue Lu, Hao Liang, Wenzhuo Li, Qi Wei, Mengling Pan, Xin Wen, Xingzhi Wang, Wei Zhou, Danni Yu, Hao Wang, Ni Yin, Hao Chen, Hansheng Li, Ting Pan, Mingyu Ma, Gaoqi Liu, Wenjia Zhou, Zhenhuang Su, Qi Chen, Fengjia Fan, Fan Zheng, Xingyu Gao, Qingqing Ji, Zhijun Ning

**Affiliations:** School of Physical Science and Technology, ShanghaiTech University, Shanghai 201210, China; School of Physical Science and Technology, ShanghaiTech University, Shanghai 201210, China; School of Physical Science and Technology, ShanghaiTech University, Shanghai 201210, China; School of Physical Science and Technology, ShanghaiTech University, Shanghai 201210, China; School of Physical Science and Technology, ShanghaiTech University, Shanghai 201210, China; School of Physical Science and Technology, ShanghaiTech University, Shanghai 201210, China; School of Physical Science and Technology, ShanghaiTech University, Shanghai 201210, China; School of Physical Science and Technology, ShanghaiTech University, Shanghai 201210, China; Department of Modern Physics, University of Science and Technology of China, Hefei 230026, China; School of Physical Science and Technology, ShanghaiTech University, Shanghai 201210, China; School of Physical Science and Technology, ShanghaiTech University, Shanghai 201210, China; School of Physical Science and Technology, ShanghaiTech University, Shanghai 201210, China; i-Lab, CAS Key Laboratory of Nanophotonic Materials and Devices, Suzhou Institute of Nano-Tech and Nano-Bionics, Suzhou 215123, China; School of Physical Science and Technology, ShanghaiTech University, Shanghai 201210, China; School of Physical Science and Technology, ShanghaiTech University, Shanghai 201210, China; School of Physical Science and Technology, ShanghaiTech University, Shanghai 201210, China; School of Physical Science and Technology, ShanghaiTech University, Shanghai 201210, China; School of Physical Science and Technology, ShanghaiTech University, Shanghai 201210, China; School of Physical Science and Technology, ShanghaiTech University, Shanghai 201210, China; Shanghai Synchrotron Radiation Facility (SSRF), Shanghai Advanced Research Institute, Chinese Academy of Sciences, Shanghai 201204, China; i-Lab, CAS Key Laboratory of Nanophotonic Materials and Devices, Suzhou Institute of Nano-Tech and Nano-Bionics, Suzhou 215123, China; Department of Modern Physics, University of Science and Technology of China, Hefei 230026, China; School of Physical Science and Technology, ShanghaiTech University, Shanghai 201210, China; Shanghai Synchrotron Radiation Facility (SSRF), Shanghai Advanced Research Institute, Chinese Academy of Sciences, Shanghai 201204, China; School of Physical Science and Technology, ShanghaiTech University, Shanghai 201210, China; School of Physical Science and Technology, ShanghaiTech University, Shanghai 201210, China

**Keywords:** perovskite solar cells, field effect transistors, heterojunction

## Abstract

Enhancing the quality of junctions is crucial for optimizing carrier extraction and suppressing recombination in semiconductor devices. In recent years, metal halide perovskite has emerged as the most promising next-generation material for optoelectronic devices. However, the construction of high-quality perovskite junctions, as well as characterization and understanding of their carrier polarity and density, remains a challenge. In this study, using combined electrical and spectroscopic characterization techniques, we investigate the doping characteristics of perovskite films by remote molecules, which is corroborated by our theoretical simulations indicating Schottky defects consisting of double ions as effective charge dopants. Through a post-treatment process involving a combination of biammonium and monoammonium molecules, we create a surface layer of n-type low-dimensional perovskite. This surface layer forms a heterojunction with the underlying 3D perovskite film, resulting in a favorable doping profile that enhances carrier extraction. The fabricated device exhibits an outstanding open-circuit voltage (*V*_OC_) up to 1.34 V and achieves a certified efficiency of 19.31% for single-junction wide-bandgap (1.77 eV) perovskite solar cells, together with significantly enhanced operational stability, thanks to the improved separation of carriers. Furthermore, we demonstrate the potential of this wide-bandgap device by achieving a certified efficiency of 27.04% and a *V*_OC_ of 2.12 V in a perovskite/perovskite tandem solar cell configuration.

## Teaser

Effective electrical doping of perovskite caused by remote molecular dopants are precisely characterized and clarified, based on which a 3D-2D heterojunction structure is fabricated to achieve a high efficiency wide bandgap perovskite solar cell, and an all perovskite tandem solar cell with efficiency over 27%.

## INTRODUCTION

Metal halide perovskites have emerged as the most promising candidates for next-generation photovoltaic materials due to their exceptional optoelectronic properties, such as high carrier mobility, large absorption coefficient and long carrier lifetime [1]. In recent years, various methods, such as composition manipulation [2–4], defect passivation [5,6], device structure optimization [7,8] and carrier transporting layer engineering [9–11], have been devoted to improving the performance of perovskite solar cells. In addition, all-perovskite-based tandem solar cells have shown promise as important architectures for enhancing efficiency beyond the Shockley-Queisser limit.

The establishment of high-quality junctions with strong interfacial electric fields based on effective electrical doping is an important approach to enhance the performance of semiconductor devices [12,13]. Several strategies have been proposed to manipulate the doping properties of perovskite films [14,15]. One involves tuning of the stoichiometric ratio of ions in the perovskite film to control its carrier polarity [16], allowing for slight adjustment of the doping density, typically on the order of ∼10^13^ cm^−3^ [17]. Another strategy involves cation doping with different valence states [18], which typically leads to deep-energy-level defects responsible for severe carrier recombination. Defect passivation using remote molecular additive capsaicin has been explored to manipulate the electrical doping of perovskite films as well, but the achieved doping densities remain around 10^13^ cm^−3^ [19]. Such low doping density can barely afford a strong electric field to effectively prompt carrier extraction [20].

The application of remote molecules such as diammonium molecules or strong molecular electron donors/acceptors has been shown to induce changes of the Fermi level based on ultraviolet photoemission spectroscopy (UPS) measurements [21–23], which were then correlated to n-type or p-type doping [24]. However, the work function is influenced by the surface dipoles introduced by adsorbed molecules and ions. Hence the movement of the surface Fermi level is generally ascribed to the formation of the surface dipole moment. Recently, field-effect transistors (FETs) have been exploited for characterizing perovskite films in terms of their carrier polarity and density. Nonetheless, the measurement of electrical doping based on remote molecules is rarely reported. The soft perovskite lattice undergoes severe ion motion with applied bias, which, in mixture with carrier transport, poses challenges for further exploring the doping effect of additive treatments [25,26].

Overall, in order to form and verify high-quality junctions in perovskite solar cells by electrical doping, the following challenges have to be addressed: (i) the preparation method of perovskite films with steep doping profiles for suppressing carrier recombination is still to be developed [27–30]; (ii) an effective characterization method to measure the electrical doping density of various perovskite films is still to be established; (iii) the mechanism for electrical doping of perovskite films by remote molecular additives still needs to be clarified.

In this study, we explored monoammonium and diammonium salts as surface additives in order to manipulate the electrical doping profile and band structure of the perovskite film surface. Using a FET structure with interdigitated arrayed electrodes and applying pulsed gate voltages, we suppressed the otherwise significant hysteresis in the transfer curves and successfully characterized the carrier type and density of wide-bandgap perovskite thin films. By treating the films with diammonium salts, we achieved a carrier density of up to ∼10^16^ cm^−3^. Theoretical simulations clarified that the formation of double ion EDA_Pb+I_ Schottky defects could give rise to n-type electrical doping. The combination of diammonium and large monoammonium molecules further induced the formation of an n-type low-dimensional structure that reduced the interface barrier for carrier extraction and simultaneously suppressed the interface carrier recombination. Leveraging the high-quality junction between the intrinsic perovskite film and the surface n-type structure, our wide-bandgap (1.77 eV) perovskite solar cells exhibit a remarkable open-circuit voltage (*V*_OC_) up to 1.34 V, resulting in an efficiency of 20.37% and a certified efficiency of 19.31%. Moreover, the rapid separation of carriers prevents electron accumulation at the interface, significantly enhancing the operational stability of the devices. To demonstrate the potential of our approach, we fabricated an all-perovskite tandem solar cell by integrating a tin-lead mixed narrow-bandgap solar cell, which achieved the best efficiency up to 27.2% with a voltage of 2.12 V and a certified efficiency of 27.04%.

## FILM FABRICATION AND STRUCTURE CHARACTERIZATION

**Figure 1.**
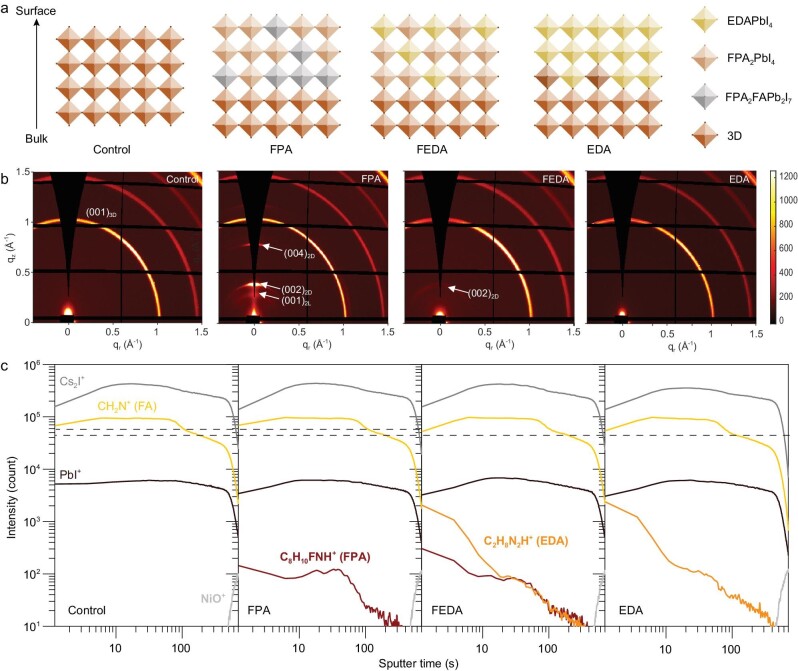
Structure characterizations. (a) Schematic of perovskite structures with EDA-, FPA- and FEDA-treated surfaces. (b) GIWAXS patterns and (c) ToF-SIMS of control, FPA, FEDA and EDA films. 2L means a two-layer structure and 2D is a two-dimensional structure.

Methylammonium (MA)-free perovskite (FA_0.8_Cs_0.2_Pb(I_0.6_Br_0.4)3_) was used to fabricate a wide-bandgap film through a typical anti-solvent method [31,32]. For the fabrication of a surface low-dimensional structure, 3-fluorophenethylamonium iodide (FPA) was used, resulting in a sample called FPA film. Another sample, treated with ethane-1,2-diammonium iodide (EDA), is called EDA film. Additionally, a film treated with a combination of FPA and EDA in a 0.5 : 0.5 weight ratio was named FEDA film (Fig. 1a). Further details regarding film fabrication and surface treatment can be found in the supplementary data. All films exhibited smooth morphology without pinholes (Fig. S1).

Initially, we investigated the surface structure of the perovskite films using grazing-incidence wide-angle X-ray scattering (GIWAXS). The FPA film exhibits diffraction peaks from two-layer (2L) and one-layer two-dimensional (2D) structures (Fig. 1b) [33,34]. The presence of strong diffraction spots indicated the formation of highly oriented low-dimensional perovskites (LDPs) parallel to the substrate [35]. In contrast, no diffraction signals are observed in the small-*q*-value region for the EDA film, suggesting low crystallinity of EDAPbI_4_. However, the measured bandgap of the film surface confirmed the presence of EDAPbI_4_, which will be discussed below. For the FEDA film, treated with a combination of FPA and EDA, a diffraction ring arising from a 2D (n = 1) structure was observed, indicating less order.

To investigate the distribution of low-dimensional perovskite structures after post-treatment, time-of-flight secondary-ion mass spectrometry (ToF-SIMS) measurements were conducted. The composition of the film at different depths was tracked through ion etching. Based on the time taken for the decrease of lead and iodide ions, as well as the increase of NiO^+^ ions, it was estimated that etching the entire perovskite film took ∼500 seconds. FPA and EDA molecules were detected within the initial 100 seconds (Fig. 1c), indicating their presence on the film surface. The intensity of the FPA signal shows no significant decrease within the first 40 seconds, suggesting a homogeneous surface distribution. Conversely, the EDA signal decreases rapidly with increasing depth, suggesting concentration on the topmost surface. In the case of the FEDA film, both FPA and EDA were observed, and the signal-decreasing times were similar to those using individual molecules. This indicates a well-mixed distribution of EDA and FPA on the perovskite film surface, resulting in a less ordered structure. Additionally, all treated perovskite films exhibited new C–N peaks (Fig. S2), verifying the presence of these molecules on the surface, as demonstrated in Fig. 1a.

## ELECTRICAL DOPING OF PEROVSKITE FILMS

**Figure 2.**
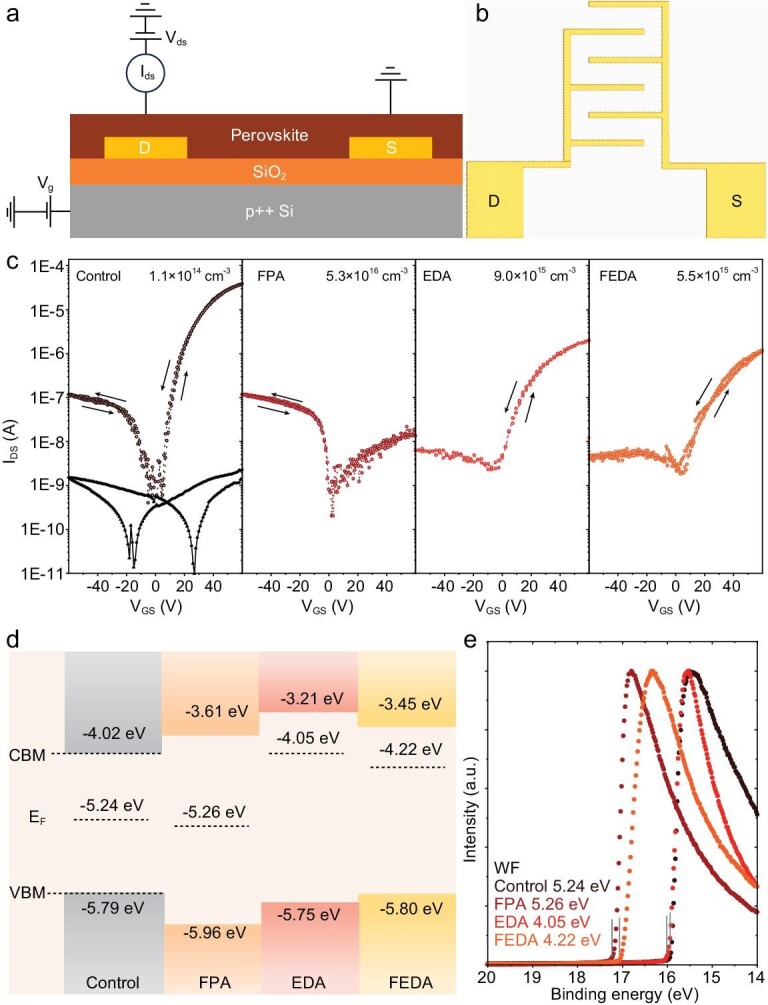
Electrical properties of the perovskite surface. (a) The electrode pattern of field-effect transistors (FETs). (b) Diagram of an interdigital electrode. (c) Transfer characteristics of control, FPA, EDA and FEDA FETs (*I*_D_, drain current; *V*_G_, gate voltage). (d) Band structures of the surface of perovskite films. VBM and CBM represent valence band maximum and conduction band minimum, respectively. (e) The UPS spectra of control, FPA, EDA and FEDA perovskite films.

To determine the electrical doping polarity and density of the thin films, we fabricated FETs (Fig. 2a) with interdigitated arrayed electrodes (IDEs) (Fig. 2b) to increase the effective channel length and the gating effect. Electrical measurements were performed by applying pulsed gate voltages to suppress any hysteresis in the transfer curves, from which the source-drain currents can be precisely extracted (see Supplementary data). As shown in Fig. 2c, the control film exhibits an increase in source-drain current in both positive and negative bias directions of the gate voltage (*V*_g_), indicating a bipolar character. The control film had an estimated carrier density of 1.1 × 10^14^ cm^−3^, featuring a nearly intrinsic semiconductor. The film processed with FPA shows an increase in current only in the negative bias direction of *V*_g_, indicating a p-type character. In contrast, both films treated with EDA and FEDA show an increased current only under positive *V*_g_ bias, indicating an n-type behavior. The carrier densities of the films treated with EDA and FEDA are derived to be 9.0 × 10^15^ cm^−3^ and 5.5 × 10^15^ cm^−3^, respectively. Although both EDA and FPA were included in the FEDA film, the electrical properties of the film seemed to be more influenced by EDA. The mechanism for n-type doping by EDA treatment will be discussed in the theoretical simulation section below.

To further confirm the electrical doping properties of the film surface, we determined the band edges of the films using UPS and inverse photoemission spectroscopy (IPES). To prevent surface reactions with oxygen and water in air, we used a movable chamber to seal the films during transport. The work function (WF) and valence band maximum (VBM) were derived based on UPS measurements (Fig. 2d), and the conduction band minimum (CBM) was calculated based on IPES spectra (Fig. S3), whose difference from VBM yielded the bandgap. The control film had a bandgap of 1.77 eV, consistent with the optical bandgap based on the absorption spectrum (Fig. S4). The FPA-treated film has a bandgap of nearly 2.35 eV, which was close to that of the 2D structure, indicating its dominant role on the film surface. The EDA-processed film surface shows a larger bandgap close to that of the one-dimensional perovskite structure, whereas the FEDA film exhibits a similar bandgap to the FPA-treated film, indicating stronger surface segregation of the 2D structure.

The WF of the film was calculated using the binding energy of the film obtained by UPS (Fig. 2e). The control film had a WF of −5.24 eV, which was close to the intermediate value of the bandgap, aligning with the nearly intrinsic character shown by FET characterization. In comparison to the control film, the FPA film shows a slightly deeper Fermi level of −5.26 eV. The EDA film shows an upshifted Fermi level of −4.05 eV, close to the CBM, indicating an n-type character. The FEDA film exhibits a Fermi level of −4.22 eV, also showing an n-type character. The change in Fermi levels of the films was further confirmed by Kelvin probe force microscopy (KPFM) measurements (Fig. S1). Compared to the control film, the surface potential is downshifted for the FPA film, while elevated for the FEDA and EDA films, consistent with the UPS results. The shifts of the Fermi level from both UPS and KPFM agree well with the FET measurements, suggesting the predominant role of electrical doping achieved by surface treatment.

To confirm that the n-type doping was caused by EDA treatment, we investigated the influence of EDA concentration on the WF. As shown in Fig. S5, the Fermi level with respect to vacuum level gradually upshifted as the EDA concentration was increased. For example, the Fermi level was −4.97 eV when the EDA concentration was 0.25 mg mL^−1^, and it increased to −3.96 eV with an EDA concentration of 0.75 mg mL^−1^. We tested another two molecules similar to EDA, namely, propane-1,3-diammonium iodide (PDA) and hexane-1,6-diammonium iodide (HAD), as shown in Fig. S6. The films treated with these molecules also show upshifted Fermi levels, indicating the universal doping functionality with diammonium salts. It should be noted that although EDA and phenylethyl amine were combined for surface treatment previously [36,37], they were only used for defect passivation.

**Figure 3.**
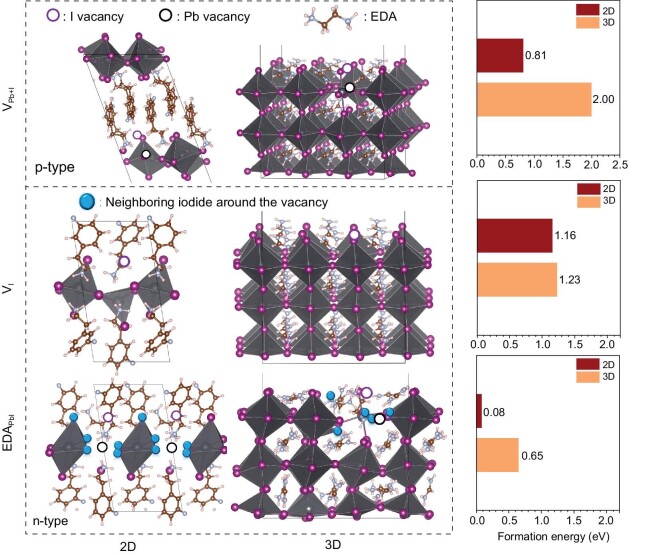
Density functional theory (DFT) simulation. p-type defects in 2D and 3D structures. The schematic of 2D and 3D structures and the defect formation energies calculated by the DFT model.

Theoretical simulations were then performed to understand the mechanism of n-type doping by EDA. The formation energies of several types of defects that could lead to n-type doping in 2D or 3D perovskites were calculated, including iodide vacancy (*V*_I_), and substitution of Pb+I by EDA (EDA_Pb+I_). As shown in Fig. 3, the formation energies of EDA_Pb+I_ ($E_{{\mathrm{ED}}{{{\mathrm{A}}}_{{\mathrm{Pb + I}}}}}^{\mathrm{f}}$) for both 2D and 3D structures were significantly lower than that of *V*_I_ ($E_{{{{\mathrm{V}}}_{\mathrm{I}}}}^{\mathrm{f}}$), indicating the prevalence of EDA_Pb+I_ defects to account for the n-type doping. The simulated low formation energy of n-type defects provides a reasonable explanation for the Fermi level change caused by EDA or similar diammonium molecules. We also simulated the formation energy of p-type *V*_Pb+I_ defects for 2D and 3D perovskite (Fig. 3). The formation energy of the *V*_Pb+I_ defect is 0.81 eV for the 2D structure, much less than that of the 3D counterpart (2.0 eV). This indicates that it is easy to form p-type defects for a 2D perovskite structure. Note that $E_{{\mathrm{ED}}{{{\mathrm{A}}}_{{\mathrm{Pb + I}}}}}^{\mathrm{f}}\! < E_{{{{\mathrm{V}}}_{{\mathrm{Pb + I}}}}}^{\mathrm{f}}$ holds for both the 2D and the 3D cases (Fig. 3), which rationalizes the overwhelming n-type doping enabled by EDA_Pb+I_ defects.

## CARRIER DYNAMIC CHARACTERIZATION

**Figure 4.**
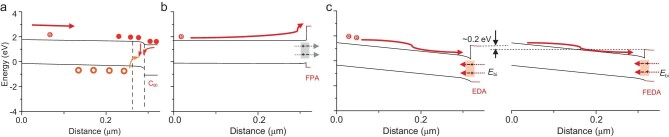
Band diagrams of perovskite films. (a) Schematic illustration of the energy band of the control group. (b and c) Schematic illustration of the formation of the heterojunction. *E*_bi_, built-in electric field at the junction.

We then simulated the band diagram across the 3D bulk structure and the low-dimensional surface structure based on the measured band edge positions and carrier densities [38–41]. For the FPA-treated film, the CBM is significantly higher than that of the 3D structure. Additionally, the larger WF of the surface 2D structure causes a slight upward bending in the 3D side, resulting in a large barrier for carrier transport from the 3D structure to the carrier transporting layer (Fig. 4a and b). In contrast, the smaller WF of the EDA-treated surface leads to a significant downward band bending that effectively reduces the barrier for carrier transport. In comparison to the EDA film, the deeper conduction band of the FEDA film reduces the barrier even further by ∼0.2 eV. This decrease in barrier height promotes the extraction of carriers to the carrier transporting layer (Fig. 4c). We note that even though an additional barrier exists in the surface-treated perovskite films, carrier ‘sweeping’ by the favorable band bending, together with the defect passivation effect, has provided a net promotion for carrier extraction, highlighting the vital role of electrical doping on the surface.

**Figure 5.**
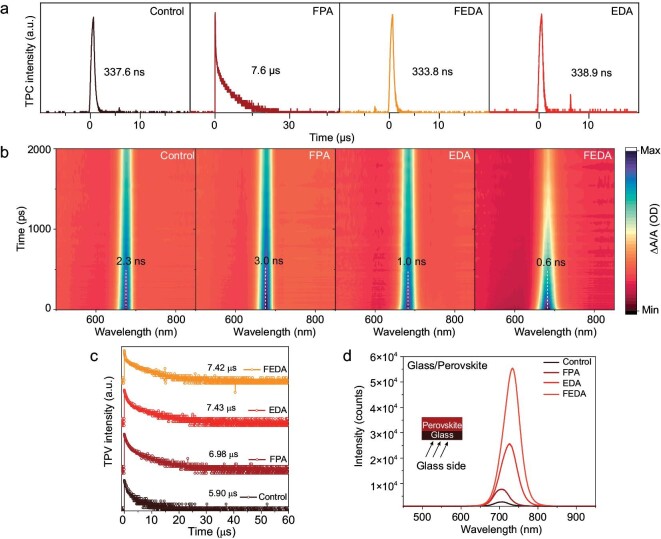
Carrier dynamic process of perovskite films. (a) Transient photocurrent curves of the devices under the short-circuit condition. (b) Transient absorption spectroscopy (TAS) spectra of perovskite films. (c) The transient photovoltage (TPV) of films with different conditions. (d) Photoluminescence spectra of films with different conditions.

Next, we investigated the carrier transport properties of the films in a solar cell device. The charge extraction time was tested using transient photocurrent (TPC) measurement under short-circuit conditions. The charge transport time substantially increased from 337.6 ns to 7.6 μs after FPA treatment (Fig. 5a). The much longer carrier lifetime in the FPA device can be attributed to the large interface barrier that inhibits electron extraction. In contrast, despite the presence of a low-dimensional surface structure, the EDA and FEDA films exhibit similar carrier lifetimes as the control sample, thanks to the decrease in barrier resulting from band bending at the interface. The EDA-processed film shows a slightly longer carrier lifetime compared to the FEDA-processed film due to a higher barrier. Transient absorption spectra were further explored for the carrier transfer between the perovskite film and the C_60_ layer. The FEDA film exhibits the fastest decay with a lifetime of 0.6 ns. In contrast, the FPA-processed film shows a larger decay time of 3.0 ns, indicating slower carrier transfer between perovskite and the electron transporting layer as well (Fig. 5b).

We then tested the transient photovoltage (TPV) under open-circuit conditions (Fig. 5c). The carrier lifetimes increase for all post-treated films, indicating a reduction in defect density due to the surface treatment. Additionally, we compared the photoluminescence (PL) intensity of the films fabricated on glass (Fig. 5d). The PL spectra indicate that all treated films show enhanced PL intensity, suggesting a reduction in defect density after the additive treatment, which is consistent with the TPV measurement. The PL peaks are slightly redshifted after the surface treatment, which could be ascribed to the increased stoichiometric ratio of iodide in the film.

## DEVICE PERFORMANCE

**Figure 6.**
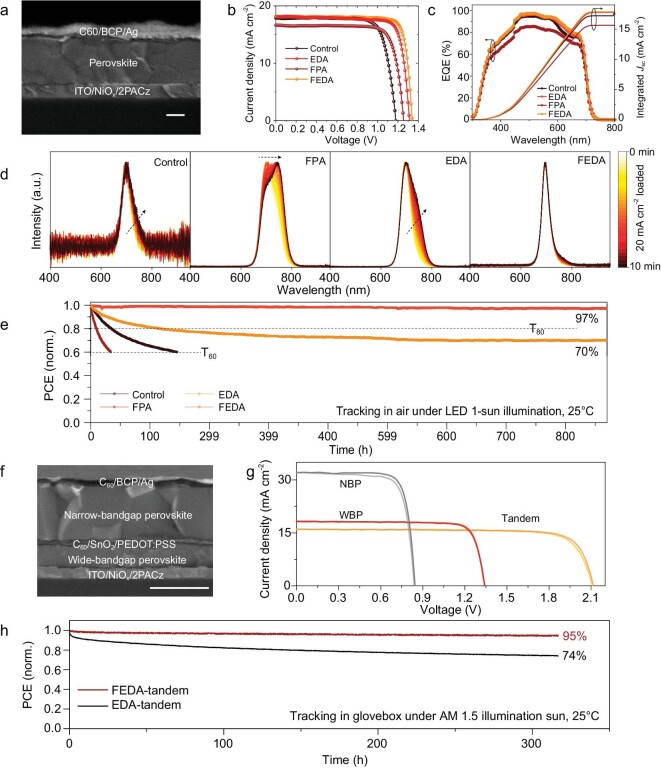
The performance of wide-bandgap perovskite solar cells and monolithic all-perovskite tandem solar cells. (a) Cross-sectional scanning electron microscopy image of a wide-bandgap device. (b and c) J–V curves (b) and EQE curves (c) of the solar cells. (d) EL-spectra tracking of devices under a current density of 20 mA cm^−2^. (e) MPP tracking of wide bandgap solar cells. (f) Cross-sectional scanning electron microscopy image of a tandem device. The scale bar is 1 μm. (g) J–V curves of the best narrow-bandgap, wide-bandgap and all-perovskite tandem solar cells. (h) MPP tracking of the tandem device based on FEDA and EDA devices.

We then investigated the performance of the surface-treated wide-bandgap (1.77 eV) solar cells using the structure of ITO/NiO_x_/2PACz/wide-bandgap perovskite/C_60_/BCP/Ag (Fig. 6a). The current density-voltage (J-V) curves of the devices are shown in Fig. 6b. Compared to the control device, solar cells treated with FPA alone exhibit a significantly increased voltage, which can be attributed to defect passivation. However, the current is reduced, likely due to an increase in the interface barrier. Both EDA- and FEDA-treated devices show enhanced current density and voltage. The higher current is likely a result of the reduced barrier and enhanced carrier extraction. The enhanced voltage can be attributed to the improved carrier extraction that reduces interface carrier recombination, as well as the better surface passivation. The FEDA device shows slightly higher current and voltage than the EDA device, likely deriving from the reduced barrier for carrier extraction. Note that the FEDA device achieves the best *V*_OC_ of 1.34 V, the highest value reported for a 1.77-eV perovskite solar cell (Figs S7 and S8, Table S1).

The best power conversion efficiency (PCE) of the FEDA device is 20.37%, one of the highest values reported for wide-bandgap solar cells, and much higher than that of the control device (Table 1). The efficiency certified by an independent lab is 19.31% (Fig. S8). The external quantum efficiency (EQE) curves of the devices are measured and shown in Fig. 6c, and the integrated current density from the EQE measurement closely matches the J_SC_ values. The FEDA device exhibits no significant loss in PCE (Fig. 6e) after 800 hours of continuous maximum power point (MPP) operation under 1-sun illumination, demonstrating superior stability compared to the FPA device. Additionally, we monitored the electroluminescence (EL) spectra under a continuous current density of ∼20 mA cm^−2^, which is equal to the J_SC_ of the device. As shown in Fig. 6d, an obvious redshift of the EL peak is observed for the FPA film, indicating severe phase segregation. This can be attributed to the larger interface barrier that induces hole accumulation and generates an extra electric field that drives ion movement. The EDA-based device shows a much-reduced peak shift, whereas the FEDA device shows no obvious EL peak shift after continuous operation, consistent with enhanced carrier extraction. Therefore, the fast carrier extraction of FEDA is also beneficial for enhancing the stability of perovskite devices.

**Table 1.** The performance of the devices measured under AM1.5 illumination. The data are average values with standard deviation from 20 devices. Parameters of the best cell are reported in brackets.

Device	PCE (%)	*V*_OC_ (V)	*J*_SC_ (mA cm^−2^)	FF (%)
Control	15.71 ± 0.39 (16.32)	1.15 ± 0.014 (1.18)	18.02 ± 0.26 (17.86)	75.66 ± 1.38 (77.42)
FPA	15.60 ± 0.42 (16.48)	1.22 ± 0.015 (1.25)	16.63 ± 0.26 (16.62)	76.62 ± 1.22 (78.92)
EDA	18.60 ± 0.54 (19.28)	1.30 ± 0.007 (1.31)	17.79 ± 0.42 (18.08)	80.16 ± 1.20 (81.11)
FEDA	19.73 ± 0.28 (20.37)	1.32 ± 0.012 (1.34)	18.21 ± 0.26 (18.16)	81.79 ± 1.20 (83.62)

To confirm the universality of this strategy, we used FEDA surface treatment for a normal-bandgap (1.55 eV) device (Fig. S9). The FEDA treatment significantly enhances the voltage from 1.09 V to 1.15 V, resulting in a PCE increase from 21.97% to 23.03%. Thus, constructing such heterojunctions could be a universal method for improving the performance of inverted structural perovskite solar cells with different bandgaps.

Based on the FEDA-treated wide-bandgap solar cells, we fabricated monolithic all-perovskite tandem solar cells combined with a ∼1.25 eV narrow-bandgap mixed Pb–Sn perovskite device (see details in Methods). The tandem device architecture is shown in Fig. 6f, with SnO_2_, Au and poly(3,4-ethylenedioxythiophene) polystyrene sulfonate (PEDOT:PSS) utilized as the interconnection recombination layer. The thickness of the Pb-Sn perovskite layer and the interconnection recombination layer are ∼1.0 μm and 50 nm, respectively (Fig. 6f). Figure 6g shows *J–V* scans of the best wide-bandgap, narrow-bandgap and tandem devices. The device performance parameters are shown in Table S2. The tandem device exhibits a high *V*_OC_ of 2.12 V, a *J*_SC_ of 16.01 mA cm^−2^ and an fill factor (FF) of 80.36%, resulting in a PCE of 27.23%, significantly higher than the control device, which has a PCE of 23.71% (Fig. S10). Under AM1.5 G 1-sun illumination, the FEDA tandem device demonstrates excellent operation stability at the MPP (Fig. 6h). We also sent one of the tandem devices for certification (Fig. S11). The certified PCE is 27.04% with a *V*_OC_ of 2.12 V, an FF of 79.98% and a *J*_SC_ of 15.90 mA cm^−2^, with negligible hysteresis. The integrated current density from the EQE curve is consistent with the *J*_SC_ value (Fig. S12). To evaluate the reproducibility of our tandem solar cells, we prepared 50 devices, and more than half of them showed efficiency higher than 26% (Fig. S13). Further scaled-up 1-cm^2^-area tandem devices demonstrated a highest PCE of 26.86% (Fig. S14).

## CONCLUSION

In this study, we investigated the use of diammonium and monoammonium molecules for post-treatment of perovskite films. Through FET measurements, we demonstrated an effective n-type doping with a carrier density of ∼10^16^ cm^−3^. By combining the diammonium and monoammonium molecules for surface treatment, we were able to create an n-type low-dimensional surface structure, which interfaced with the underlying 3D perovskite, constituting an effective heterojunction that reduced the interface barrier and suppressed carrier recombination. Consequently, we achieved a high voltage of 1.34 V for the surface-treated wide-bandgap perovskite solar cells, resulting in an efficiency of 20.37% and significantly improved operation stability. Building upon the improved photovoltage for wide-bandgap perovskites, we fabricated all-perovskite tandem solar cells with the best efficiency of 27.23% and a high voltage of 2.12 V. Furthermore, we demonstrated the applicability of this method to normal-bandgap (1.5 eV) perovskite solar cells. This work strongly indicates that molecular dopants can be effective ingredients for manipulating the doping profiles of perovskite films, whose overall density can be directly characterized by FETs. The establishment of high-quality surface junctions by additive treatment thus offers a promising avenue for enhancing the efficiency of inverted perovskite solar cells. The manipulation of the electrical doping of perovskite films presents a promising avenue to enhance the performance of perovskite-based optoelectronic devices.

## Supplementary Material

nwae055_Supplemental_File
