# Supplementary material for: Surface heterojunction based on n-type low-dimensional perovskite film for highly efficient perovskite tandem solar cells
Source: Natl Sci Rev. 2024 Feb 13;11(5):nwae055. doi: 10.1093/nsr/nwae055 (PMC10989298; doi:10.1093/nsr/nwae055)
Supplement: nwae055_Supplemental_File [file nwae055_supplemental_file.pdf]

Supplementary Materials for

Surface heterojunction based on n-type low-dimensional perovskite film for highly efficient perovskite tandem solar cells

Authors: *Xianyuan Jiang*[†1], *Qilin Zhou*[†1], *Yue Lu*[1], *Hao Liang*[1], *Wenzhuo Li*[1], *Qi Wei*[1], *Mengling Pan*[1] , *Xin Wen*[1], *Xingzhi Wang*[2] *Wei Zhou*[1], *Danni Yu*[1], *Hao Wang*[1], *Ni Yin*[3], *Hao Chen*[1], *Hansheng Li*[1], *Ting Pan*[1], *Mingyu Ma*[1], *Gaoqi Liu*[1], *Wenjia Zhou*[1], *Zhenhuang Su*[4], *Qi Chen*[3], *Fengjia Fan*[2], *Fan Zheng*[1], *Xingyu Gao*[4], *Qingqing Ji**[1], *Zhijun Ning**[1]

* Correspondence to: jiqq@shanghaitech.edu.cn; ningzhj@shanghaitech.edu.cn

Materials and Methods

Materials. Stannous Fluoride (SnF_2, 99%), Lead (II) thiocyanate ($Pb(SCN)_2$, 99.99%), Anisole (anhydrous, 99.7%), 2-Propanol (IPA, anhydrous, 99.5%), Ethanol Absolute (anhydrous, ≥99.8%), N,N-Dimethylformamide (DMF, anhydrous, 99.8%) and Dimethyl sulfoxide (DMSO, anhydrous, 99.9%) were purchased from Sigma-Aldrich;; C_{60} (>99.5%), PEDOT: PSS (CLEVIOS^{TM} PVP AI 4083), Bathocuproine (BCP, >99%, sublimed) ,3-Fluorophenethylamine iodide (FPA, 98%), Ethane-1,2-Diammonium Iodide (EDA, 98%), were purchased from Xi'an Polymer Light Technology. Methylammonium Iodide (MAI, >99.99%), Formamidinium Iodide (FAI, >99.99%), Cesium Iodide (CsI, >99.0%), Lead(II) Iodide (PbI_2, 99.99%), Lead(II) Bromide ($PbBr_2$, >98.0%), and [2-(9H-Carbazol-9-yl)ethyl]phosphonic Acid (2PACz, >98.0%) were purchased from Tokyo Chemical Industry (TCI). All reagents were used directly without being purified. NiO_x nanoparticle and SnI_2 were prepared according to our previous work [1, 2].

Perovskite precursor solution. To make wide-bandgap perovskite films, 1.2 M $FA_{0.8}Cs_{0.2}Pb(I_{0.6}Br_{0.4})_3$ perovskite precursor solution was prepared by dissolving CsI, FAI, $PbBr_2$ and PbI_2 in mixed solvents of DMF and DMSO with a volume ratio of 3:1. The precursor solution was stirred at 60 ^{o}C for 2 hours before use. To make narrow-bandgap perovskite films, 2.0 M $Cs_{0.05}FA_{0.7}MA_{0.25}Pb_{0.5}Sn_{0.5}I_3$ precursor solution was prepared by dissolving FAI, MAI, CsI, PbI_2, SnI_2, and SnF_2 in mixture solvent (DMSO: DMF = 1:3 v/v). The precursor solution was then stirred at room temperature for 2 hours. To make normal bandgap (1.5 eV) perovskite films, a 1.4 M perovskite precursor comprised of CsI, FAI, PbI_2, and CsSCN in the molar ratio of 0.03: 0.97: 1: 0.02 were added in a mixed solvent (DMSO: DMF =1:4 v/v) and stirred at 50 ^{0} C for 2 hours before use. All precursors were filtered with 0.22 μm PTFE filters before using.

Single junction wide-bandgap perovskite solar cells fabrication. NiO_x nanocrystal (10 mg mL^{-1} in H_2O and IPA mixed solvent with volume ratio of 3:1) layers were first spin-coated on ITO substrates at 3,000 rpm for 25 s in air without annealing, Subsequently the substrates were immediately transferred to the glovebox. 2PACz solution (0.5 mg mL^{-1}) in ethanol was spin-coated on the NiO_x film at 3,000 rpm for 25 s and then annealed at 100 ^oC for 10 min. For the perovskite film fabrication, the substrate was spun at 4,000 rpm for 35 s with an acceleration of 1,000 rpm, 150 μL Anisole was dropped onto the substrate during the last 5 s of the spinning. The substrates were then transferred onto a hotplate and heated at 100 ^oC for 10 min. The organic salts surface treatment solutions were prepared by dissolving FPA, or FPA and EDA, or EDA in IPA with different concentrations. The optimal concentration of EDA used in devices was 1 mg mL^{-1}. The surface treatment was finished by depositing 100 μL organic salts solution onto the perovskite film surface at a spin rate of 3,000 rpm for 25 seconds with a 1,000 rpm s^{-1} acceleration. The film was then annealed at 100 ^oC for 10 min. After cooling down to room temperature, the substrates were transferred to the evaporation system and a 23-nm-thick C_{60} film was deposited on top by thermal evaporation at a rate of 0.2 A s^{-1}, followed by evaporation of 8 nm BCP at a rate of 0.2 A s^{-1}, then finished by thermal evaporation of 100-nm-thick Ag electrode.

Monolithic 2-T all-perovskite tandem solar cell fabrication. The wide-bandgap perovskite solar cell fabrication was completed as described above until the deposition of C_{60}. Then followed by atomic layer deposition of SnO_2, 20 nm SnO_2 was deposited at 90 ℃ using precursors of tetrakis(dimethylamino) tin (iv) (99.9999%) and deionized water as the precursors. And 1 nm layer of Au was deposited by thermal evaporation. Next, PEDOT: PSS (diluted at a 1:1 volume ratio in IPA) was spin-coated onto the wide-bandgap subcell at 4,000 rpm for 30 s and annealed at 120 ℃ for 10 min. After cooling, the substrates were immediately transferred to a glovebox for the deposition of perovskite films. The perovskite films were deposited with a two-step spin-coating procedure: (1) 1,000 rpm for 10 s with an acceleration of 200 rpm s^{-1}, (2) 4,000 rpm

for 45s with an acceleration of 1,000 rpm s^{-1}, 250 μL anisole was dropped onto the substrate during the second spin-coating step at 5 s before the end of the procedure. The substrates were then treated on hotplate at 60 °C for 30 s, then 100 °C for 7 min. Post-treatment with EDA was carried out by spin-coating a solution of 0.8 mg mL^{-1} EDA in IPA at 4,000 rpm for 25 s, followed by annealing at 100 ℃ for 5 min. After cooling, the substrates were transferred to thermal evaporation system, 23 nm C_{60}, 8 nm BCP and 100 nm Ag were sequentially deposited on top of the perovskite layer by thermal evaporation.

Normal bandgap (1.5 eV) perovskite solar cell fabrication. The processes of NiO_x and 2PACz layers are same as described above. The precursor was spin coated on the substrate at 1,500 and 6,000 rpm for 15 s and 25 s, respectively. During the second stage, 150 μL anisole was dropped onto the surface at the final 10 s, and the substrate was annealed at 100 °C for 10 min. The surface treatment was finished by depositing 100 μL organic salts solution (FEDA, 1mg mL^{-1}) onto the perovskite film surface at a spin rate of 3,000 rpm for 25 s with a 1,000 rpm s^{-1} acceleration. After cooling, the substrates were transferred to thermal evaporation system, 23 nm C_{60}, 8 nm BCP and 100 nm Ag were sequentially deposited on top of the perovskite layer by thermal evaporation.

PL. To avoid environmentally induced degradation, perovskite films were encapsulated by quartz glass and UV-glue. PL spectra were detected using a spectrofluorometer (Fluorolog; HORIBA FL-3) with an exciting wavelength of 400 nm.

GIWAXS. GIWAXS measurement was performed by employing a beam energy of 10 keV and a PILATUS detector at the BL17B1 beamline of Shanghai Synchrotron Radiation Facility (SSRF), Shanghai, China. GIWAXS patterns were acquired using incident angle of 0.4°, and the sample located about 330 mm away from the detector (Pilatus). Using the GIXGUI Matlab toolbox to correct and reshape raw patterns[3].

FET characterization. (1) Device Fabrication. A photoresist layer was patterned by photolithography on a p++ Si/SiO_2(300 nm) substrate, which was rendered hydrophilic in advance by oxygen plasma. Subsequently, the photolithographic patterns were converted into metal electrodes by electron-beam evaporation and lift-off of a 5 nm Cr/30 nm Au layer. The perovskite precursor solution was spin-coated onto the substrate with pre-fabricated electrodes and annealed for follow-up device measurement. (2) Electrical measurements. The gate voltage was applied under a sweep rate of 0.6 V/s, using 10-ms-wide pulses generated by the Keysight B2912B source meter. The measurement was performed at room temperature in a vacuumed probe station with pressure of ~0.5 Pa. Carrier concentration (n) was calculated by $n = \frac{\sigma C_{\mathrm{ox}} w V_{\mathrm{ds}}}{e g_{\mathrm{m}} l}$, where σ is the source-drain conductivity, e is the elementary charge (1.6×10^{-19} C), g_{m} is the transconductance, l is the channel length (100 μm), C_{ox} is the oxide capacitance per unit area (11.5 nF/cm^2), w is the effective channel width (1 mm), and V_{ds} is the bias voltage (60 V).

UPS. UPS measurement was obtained by Thermo Fisher ESCALAB 250XI. All perovskite films fabricated on ITO/NiOx/2PACz substrate and were transferred from glovebox to ESCALAB 250XI chamber using a portable gas-tight capsule. A sample bias of -10 V was applied for UPS acquisition.

IPES: Inverse photoemission spectroscopy (IPES) measurement was performed using a customized ULVAC-PHI LEIPS instrument with Bremsstrahlung isochromatic mode.

KPFM and AFM. The amplitude-modulation KPFM was operated combined with a Cypher S atomic force microscopy (AFM; Asylum Research, Oxford Instruments) and a HF2LI Lock-in amplifier (Zurich Instruments) in N_2-filled glovebox. The resonance frequency ω0 and spring constant of AFM conducting tips are ~127 kHz

and 5.0 Nm^{-1}, respectively.

TPV and TPC. A 640 nm diode laser was used to modulate the V_{OC} on top of a constant light bias. The pulse duration was set to 1 μs and the repetition rate to 50 Hz by the function generator of the oscillator. The digital oscilloscope recorded the data induced by the light perturbation, using 1 MΩ input impedance for the TPV measurement and 50 Ω impedance for TPC measurement.

EL. The current density–luminance-luminescence (*J-V-L*) characteristics and EQE values were acquired by a Keithley 2612B source meter and a fiber integrating sphere (FOIS-1) couple with a QE Pro650 spectrometer (SpectrumTEQ-EL system, Ocean Optics). The EQE of LED can be defined as $EQE=\frac{\text{emitted photons out LED}/\text{second}}{\text{injected electrons}/\text{second}}$. The counts of photons per second are collected by an integrating sphere and a fiber spectrometer and the counts of electrons are collected with Keithley 2612B source meter. The LED devices were tested on top of the integrating sphere, and only forward light emission could be collected, which is consistent with the standard OLED characterization method. All the device test processes were performed in the N_2-filled glovebox.

SEM, UV–vis, and ToF-SIMS measurements

The surface and cross-sectional microscope images were taken by means of a scanning electron microscope (SEM, JSM-7800, JEOL). The ultraviolet-visible spectra (UV–vis) of the perovskite films deposited on NiOx/2PACz were measured using an UV-vis spectrophotometer (Agilent cary5000). ToF-SIMS analysis was carried out with a ToF-SIMS 5-100 instrument (ION-TOF GmbH, Germany).

Solar cell characterizations. The calibration of light was enabled by a KG-2 Si diode with a solar simulator (Enli Tech, Taiwan, China). A Keithley 2400 source unit was employed to obtain *J-V* curves under simulated AM1.5G solar illumination at 100

mW cm^{-2} (1 sun). For the measurement of the *J-V* curve, the solar cell was masked applying an aperture mask with an area of 0.042 cm^2. The applied bias ranges from 0 V to 1.36 V for wide-bandgap perovskite solar cell, from 0 V to 0.86 V for narrow-bandgap solar cell, from 0 V to 2.15 V for tandem solar cell at room temperature in a glovebox. No preconditioning was used before *J–V* measurements. A commercial system (Solar cell scan 100, Beijing Zolix Instruments Co., Ltd) was used to measure the EQE spectra. And the calibration of light intensity was carried out by a standard photodetector (QE-B3/S1337-1010BQ, Zolix). The light beam was chopped at 180 Hz and the response of the cell was acquired by a Stanford Research SR830 lock-in amplifier. For all-perovskite tandem solar cell, the bias illumination from highly-bright LEDs with emission FPAks of 850 and 460 nm was employed to measure the spectral response of the top and bottom subcells, respectively, respectively. EQE measurements were performed in ambient air, and no bias voltage was applied during the EQE measurements. The solar cell certification measurements were carried out by Shanghai Institute of Microsystem and Information Technology (SIMIT), Chinese Academy of Sciences.

DFT. All the DFT-based first-principles calculations are performed by using the Vienna Ab-initio Simulation Package[4, 5] (VASP) with the projector augmented wave [6] (PAW) method. Generalized-gradient approximation [7] (GGA) formulated by Perdew, Burke, and Ernzerhof (PBE) is used as the exchange-correlation functional. And the plane-wave cutoff energy is set to 400 eV. The Gamma-centered k-point mesh with a grid spacing of 2π×0.03 Å^{-1} is used for electronic Brillouin-zone integration. DFT-D2[46] method of Grimme was implemented to take into account the long-range van der Waals (vdWs) interaction. For the defect formation energy calculation, the thickness of vacuum for 3D-slab structure is set to be 15 Å and the initial 2D structure is based on reported structure. [8] The defect formation energy of $E^f_{V_I}$, $E^f_{EDA_{Pb+I}}$ are calculated as:

$$E^f_{V_I} = E_{V_I} - E_{\text{host}} + \mu_I$$

$$E^{f}_{EDA_{Pb+I}} = E_{EDA_{Pb+I}} - E_{\mathrm{host}} + \mu_{I} + \mu_{Pb} - \mu_{EDA}$$

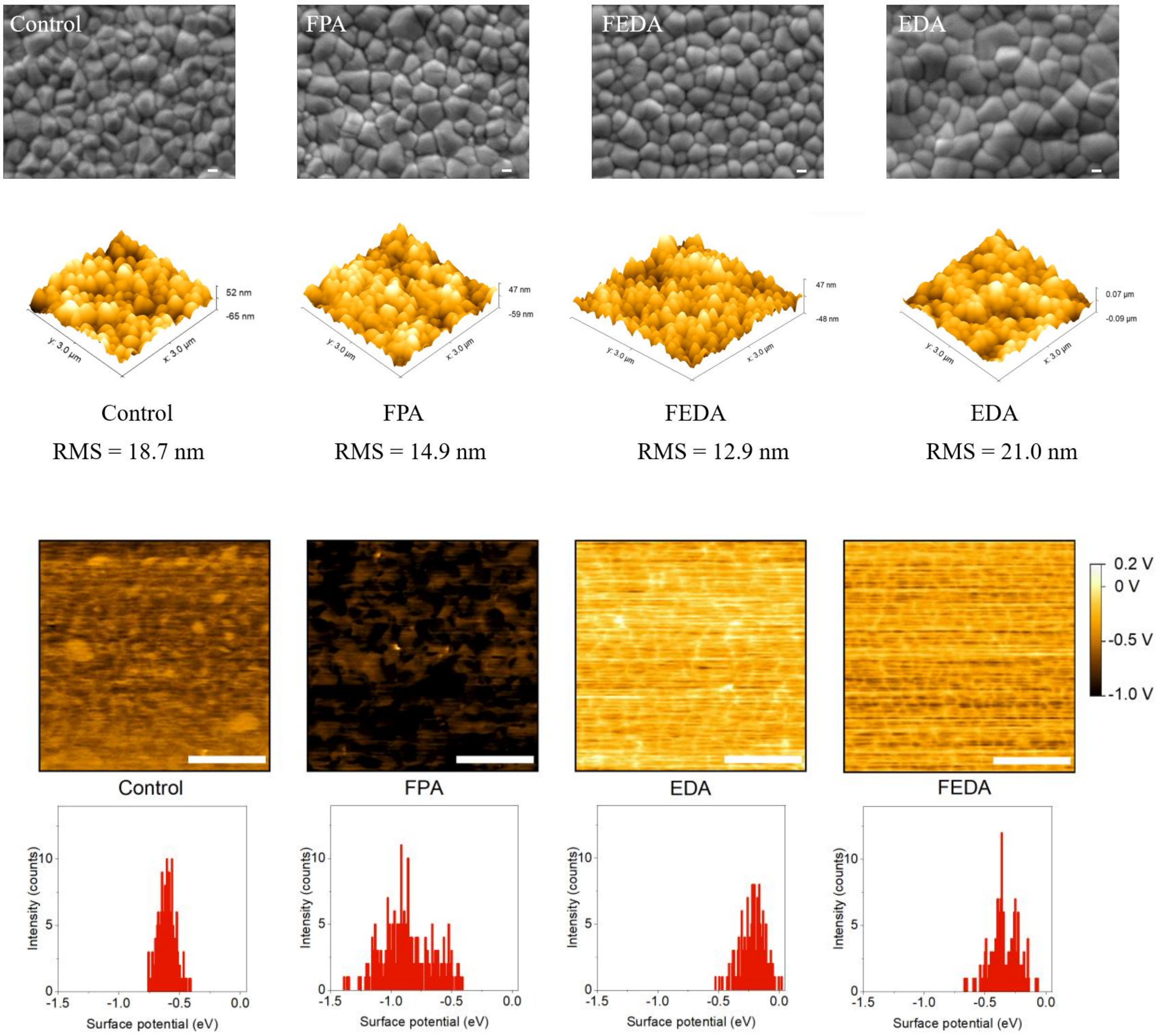

Figure S1. Perovskites film morphology. a,SEM and b,AFM images of control, FPA, FEDA, and EDA films. The scale bar in SEM is 100 nm. c,KPFM images of the surface potential of perovskite films.

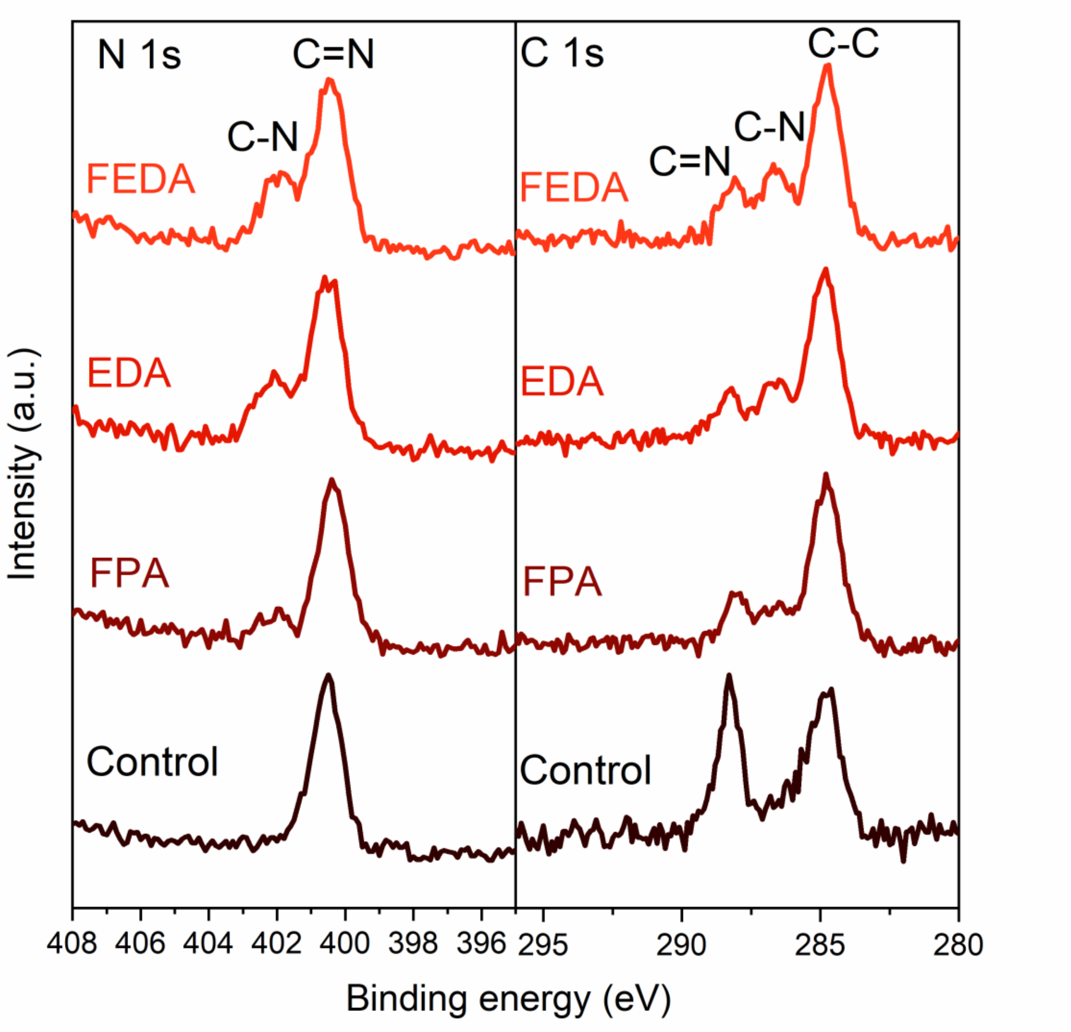

Figure S2. High-resolution N 1s spectra and C 1s spectra of pristine and treated perovskite films obtained by XPS characterization.

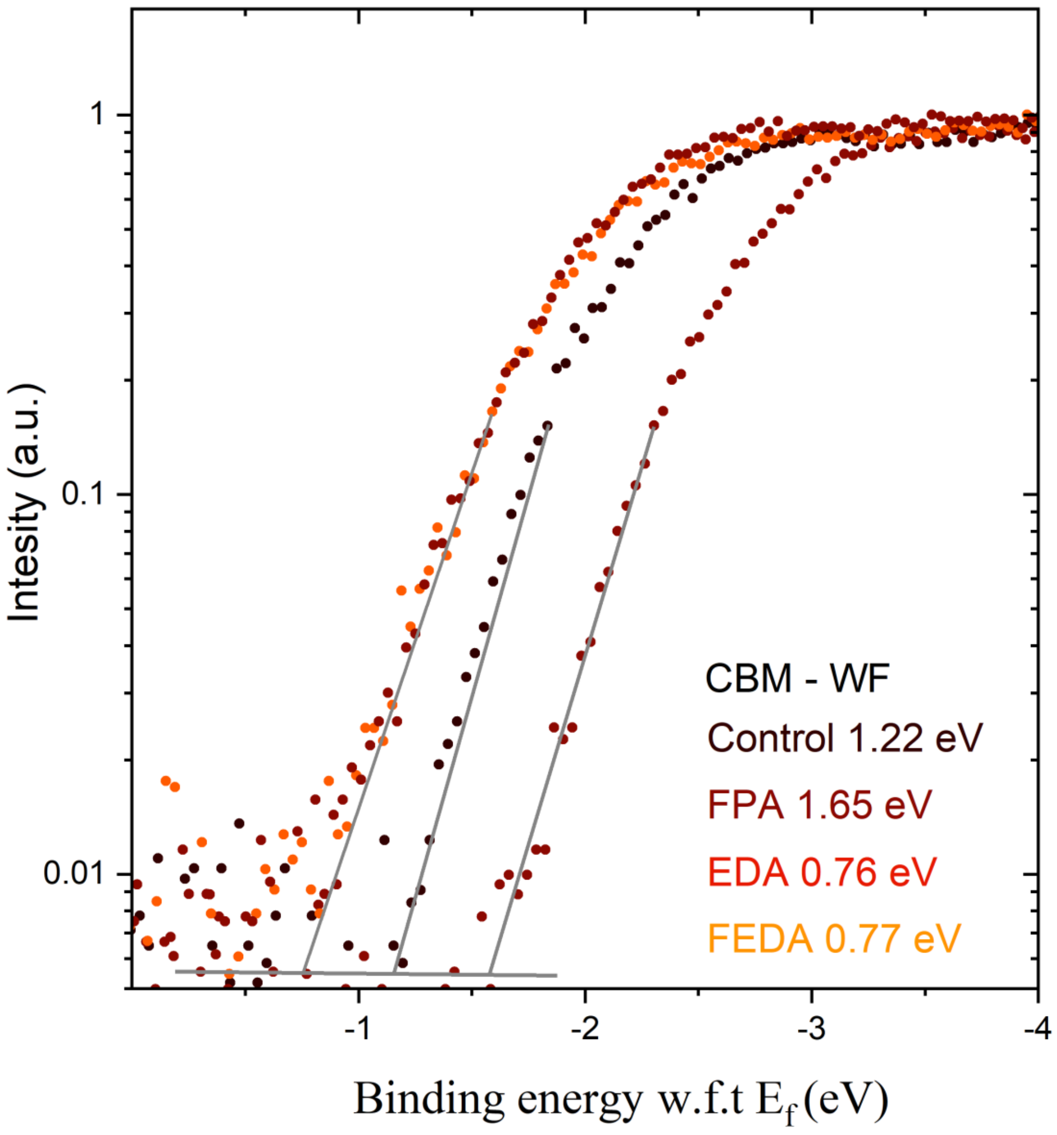

Figure S3. IPES spectra of control, FPA, EDA, and FPDA perovskite films.

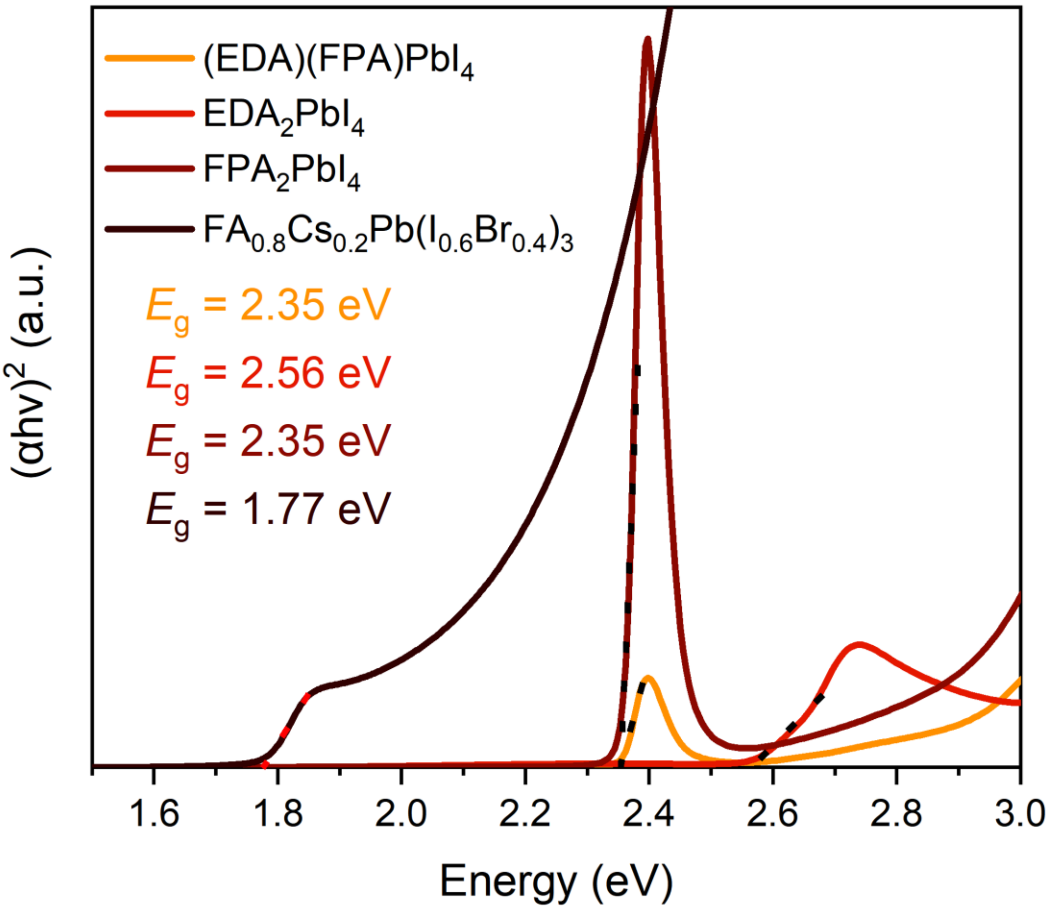

Figure S4. Tauc plots of perovskite films. Based on Tauc plots, both FPA_2PbI_4 and $(EDA)(FPA)PbI_4$ have a bandgap of 2.35 eV, while the $EDAPbI_4$ has a larger bandgap of 2.56 eV.

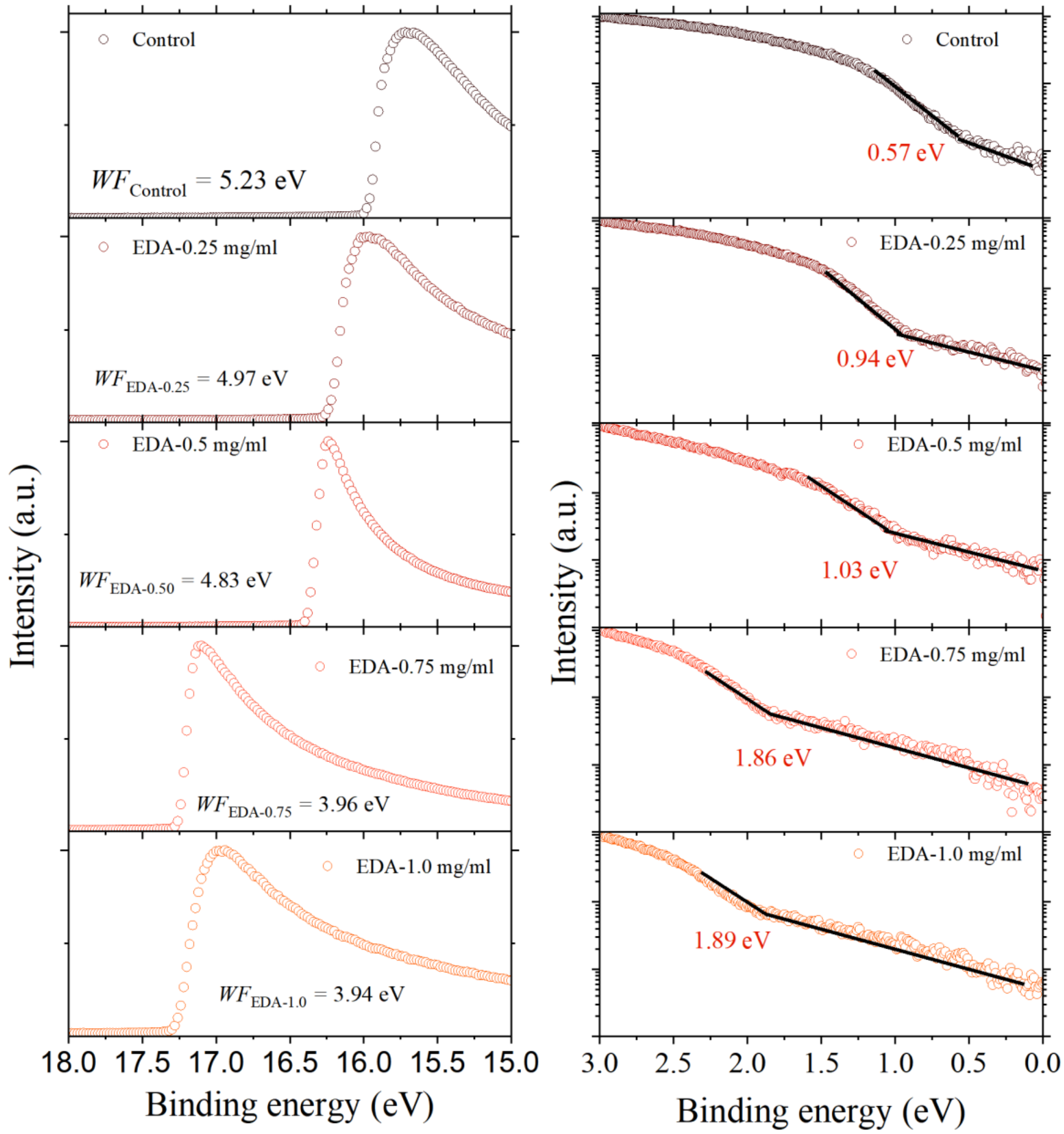

Figure S5. UPS data for the pristine and different EDA solvents post-treated films.

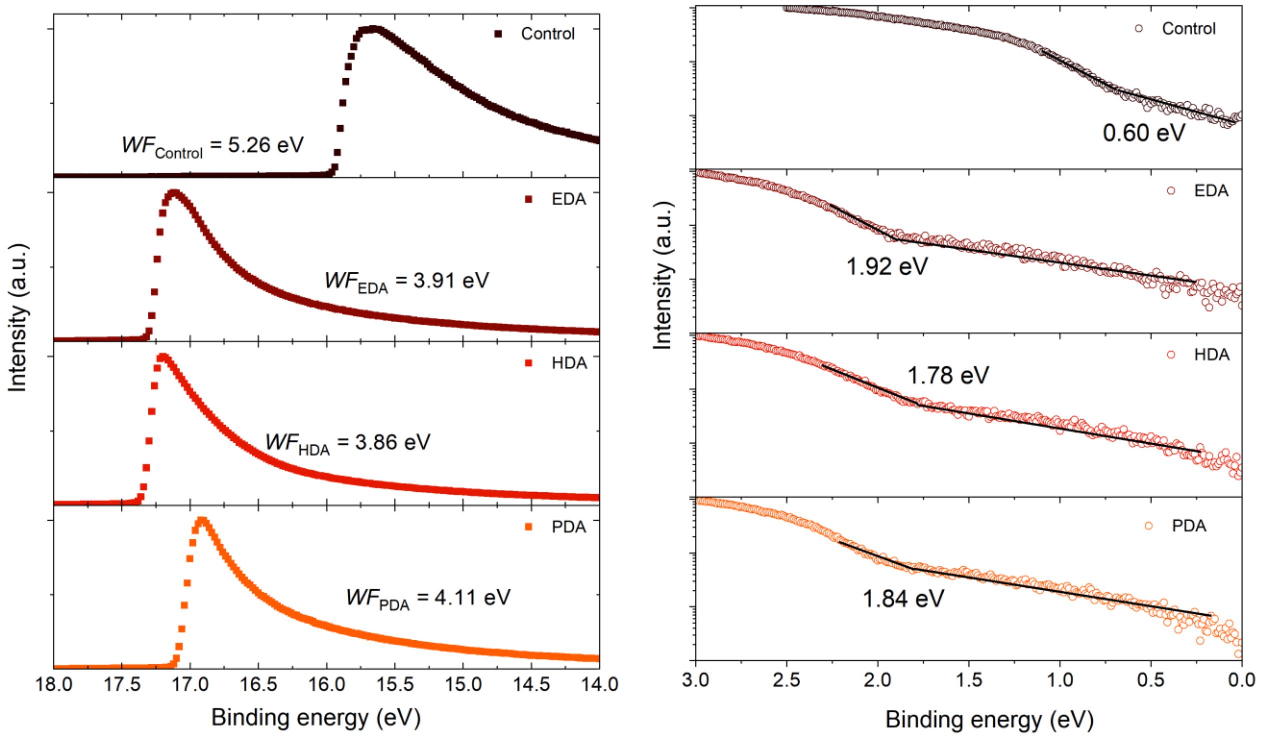

Figure S6. UPS data for the pristine and post-treated films. PDA is propane-1,3-diammonium iodide and HAD is hexane-1,6-diammonium iodide, and all post-treatment precursor concentration is 1 mg mL^{-1}. The corresponding *WF* and E_{VBM} are summarized in the inset.

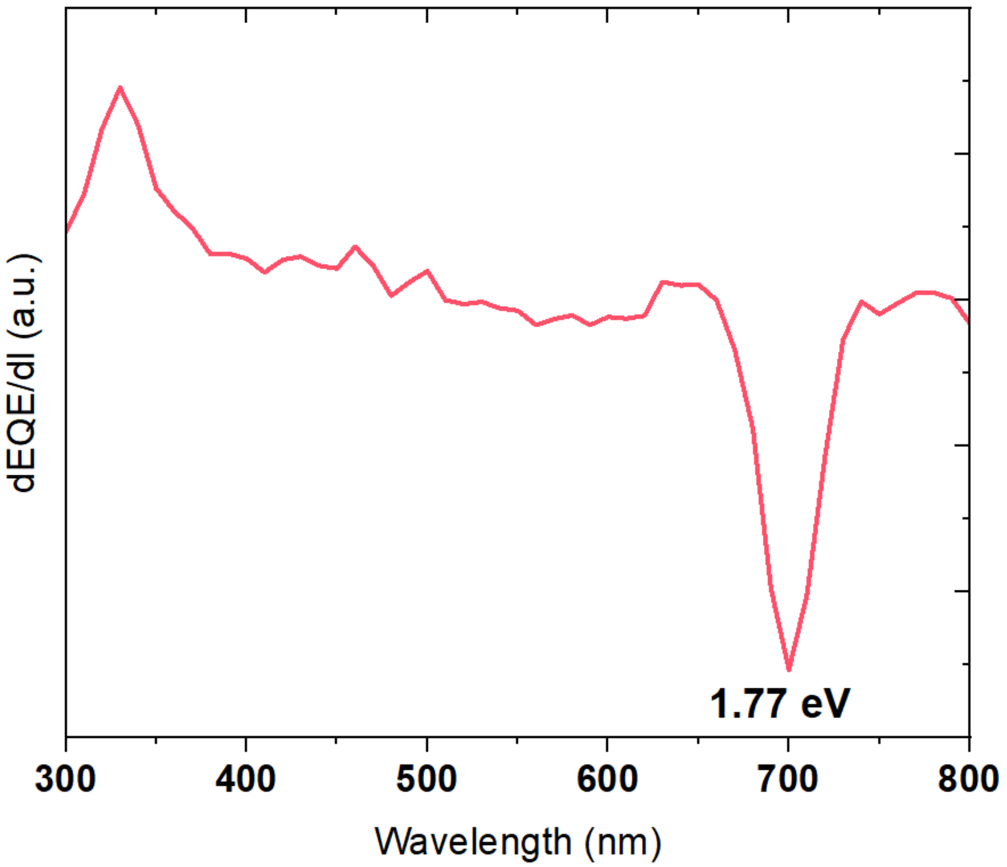

Figure S7. EQE measurement of FEDA solar cell, showing a device bandgap of 1.77 eV.

Sample Information

Sample Type	Perovskite Solar Cell
Serial No.	1-7#
Lab Internal No.	23011301-3#
Measurement Item	I-V characteristic
Measurement Environment	24.3±2.0°C, 41.7±5.0%R.H

Measurement of I-V characteristic

**Reference cell**	AK-200(Serial No.:2000041)
**Reference cell Type**	mono-Si, WPVS, calibrated by National Institute of Metrology, China (Certificate No. GXgf2022-01035)
**Calibration Value/Date of Calibration for Reference cell**	128.1mA/ Apr. 2022
**Measurement Conditions**	Standard Test Condition (STC):<br>Spectral Distribution: AM1.5 according to IEC 60904-3 Ed.3,<br>Irradiance: 1000±50W/m^2, Temperature: 25±2°C
**Measurement Equipment/ Date of Calibration**	AAA Steady State Solar Simulator (YSS-T155-2M) / July.2022<br>IV test system (ADCMT 6246) / June. 2022<br>SR Measurement System(CEP-25ML-CAS)/April.2022<br>Measuring Microscope (MF-B2017C) / July.2022
**Measurement Method**	I-V Measurement:<br>Logarithmic sweep in both directions (Voc to Isc and Isc to Voc) during one flash based on IEC 60904-1;<br>Spectral Mismatch factor was calculated according to IEC 60904-7 and I-V correction according to IEC 60891;
**Measurement Uncertainty**	Area: 1.0%(k=2); Isc: 1.8%(k=2); Voc: 1.0%(k=2);<br>Pmax: 2.5%(k=2); Eff: 2.6%(k=2)

====Measurement Results ====

	Forward Scan (Isc to Voc)	Reverse Scan (Voc to Isc)
Area[da]	4.20mm^2	
Isc	0.732 mA	0.732 mA
Voc	1.323 V	1.324 V
Pmax	0.804 mW	0.811 mW
Ipm	0.694 mA	0.698 mA
Vpm	1.158 V	1.162 V
FF	82.98 %	83.64 %
Eff	19.14 %	19.31 %

- Spectral Mismatch factor SMM_{top}=1.0103;
- Designated illumination area defined by a thin metal mask was measured by a measuring microscope.
- Test results listed in this measurement report refer exclusively to the mentioned test sample.
- The results apply only at the time of the test, and do not imply future performance.

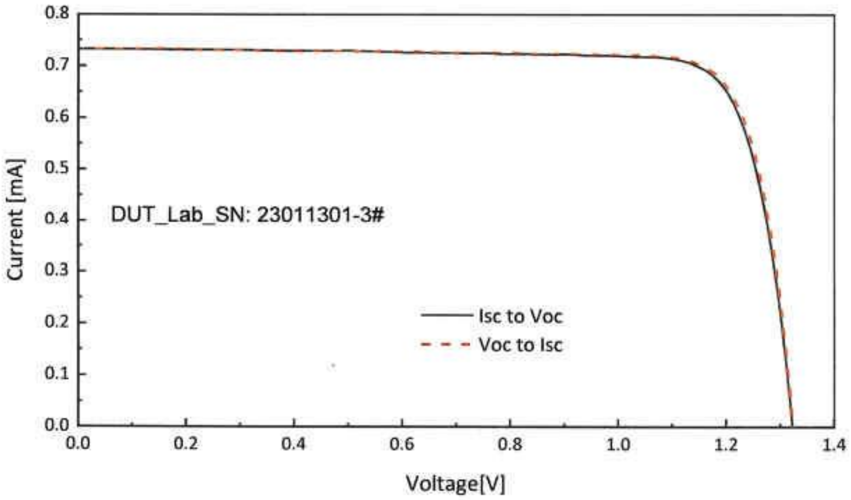

Fig.1 I-V curves of the measured sample

Report No. 23TR011303

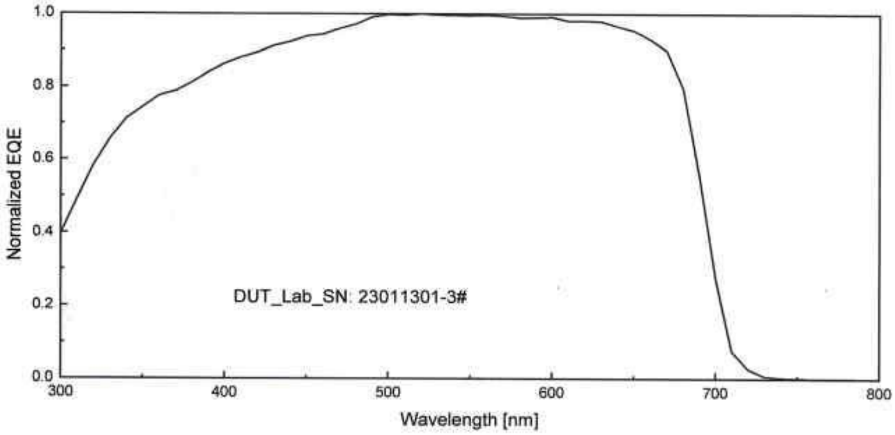

Fig.2 Normalized EQE curve of the measured sample

-------End of Report-------

Figure S8. Certification of photovoltaic performance of the wide-bandgap FEDA device measured by Shanghai Institute of Microsystem and Information Technology (SIMIT).

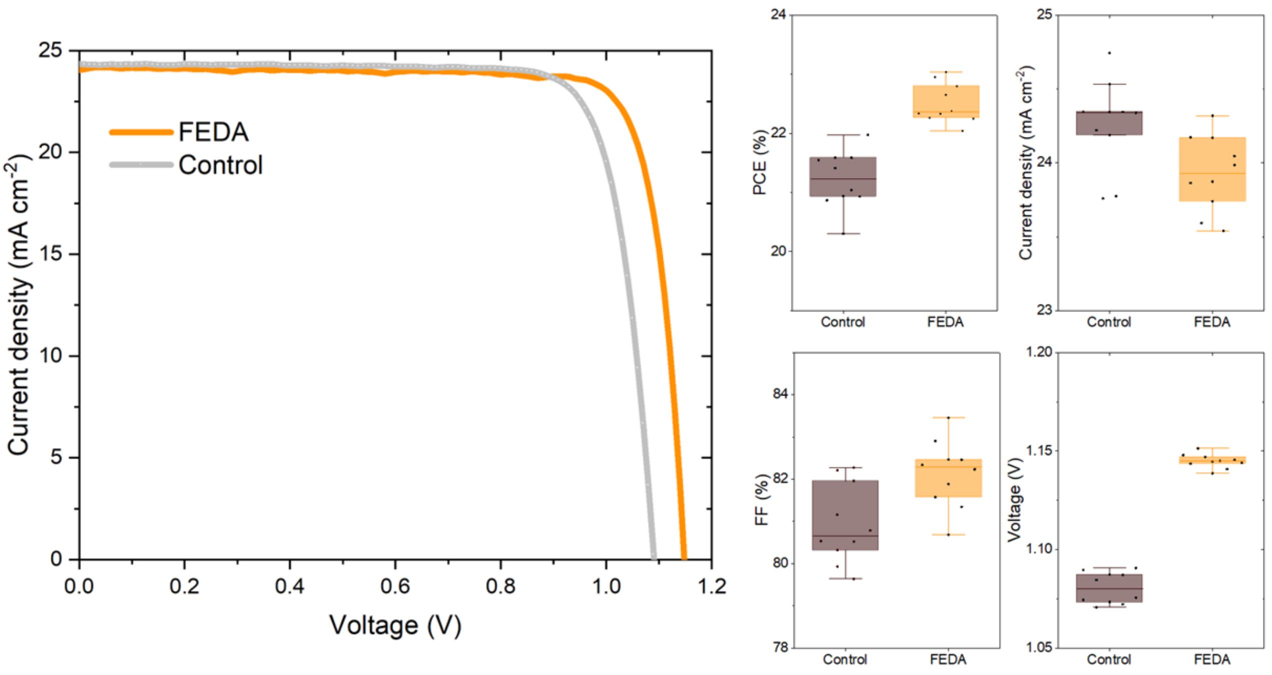

Figure S9. *J*-*V* curves and statistic parameters of the normal bandgap (1.5 eV) perovskite devices.

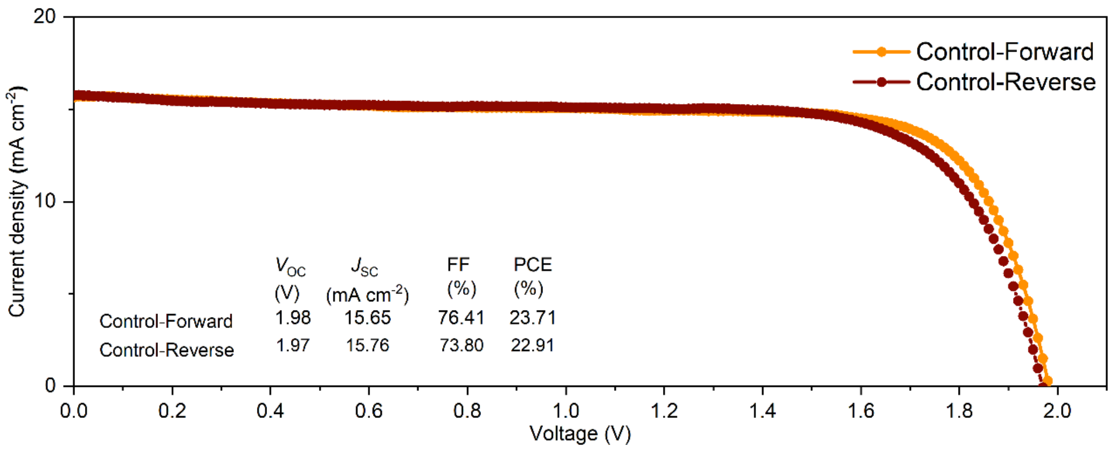

Figure S10. *J-V* curve of the tandem solar cell based on control device.

中国认可
国际互认
检测
TESTING
CNAS L8490

Test and Calibration Center of New Energy Device and Module,
Shanghai Institute of Microsystem and Information Technology,
Chinese Academy of Sciences (SIMIT)

Measurement Report

Report No. 23TR011302

**Client Name**	ShanghaiTech University, Zhijun Ning Group
**Client Address**	393 Middle Huaxia Road, Pudong, Shanghai, China
**Sample**	Perovskite/perovskite tandem solar cell
**Manufacturer**	ShanghaiTech University
**Measurement Date**	13th January, 2023

**Performed by:**	Qiang Shi *Qiang Shi*	Date: 13/01/2023
**Reviewed by:**	Wenjie Zhao *Wenjie Zhao*	Date: 13/01/2023
**Approved by:**	Zhengxin Liu *Zhengxin Liu*	Date: 13/01/2023

检测专用章

Address: No.235 Chengbei Road, Jiading, Shanghai **Post Code:**201800
E-mail: solarcell@mail.sim.ac.cn **Tel:** +86-021-69976921

The measurement report without signature and seal are not valid.
This report shall not be reproduced, except in full, without the approval of SIMIT.

Sample Information

Sample Type	Perovskite/Perovskite Tandem Solar Cell
Serial No.	2#
Lab Internal No.	23011301-2#
Measurement Item	I-V characteristic
Measurement Environment	24.3±2.0°C, 41.7±5.0%R.H

Measurement of I-V characteristic

**Reference cell**	AK-200(Serial No.:2000041)
**Reference cell Type**	mono-Si, WPVS, calibrated by National Institute of Metrology, China (Certificate No. GXgf2022-01035)
**Calibration Value/Date of Calibration for Reference cell**	128.1mA/ Apr. 2022
**Measurement Conditions**	Standard Test Condition (STC):<br>Spectral Distribution: AM1.5 according to IEC 60904-3 Ed.3,<br>Irradiance: 1000±50W/m^2, Temperature: 25±2°C
**Measurement Equipment/ Date of Calibration**	AAA Steady State Solar Simulator (YSS-T155-2M) / July.2022<br>IV test system (ADCMT 6246) / June. 2022<br>SR Measurement System(CEP-25ML-CAS)/April.2022<br>Measuring Microscope (MF-B2017C) / July.2022
**Measurement Method**	I-V Measurement:<br>Logarithmic sweep in both directions (Voc to Isc and Isc to Voc) during one flash based on IEC 60904-1;<br>Spectral Mismatch factor was calculated according to IEC 60904-7 and I-V correction according to IEC 60891;
**Measurement Uncertainty**	Area: 1.0%(k=2); Isc: 2.1 %(k=2); Voc: 1.0%(k=2);<br>Pmax: 2.6%(k=2); Eff: 2.8%(k=2)

====Measurement Results ====

	Forward Scan (Isc to Voc)	Reverse Scan (Voc to Isc)
Area[da]	4.20mm^2	
Isc	0.668 mA	0.668 mA
Voc	2.124 V	2.126 V
Pmax	1.117 mW	1.136 mW
Ipm	0.617 mA	0.619 mA
Vpm	1.811 V	1.836 V
FF	78.75 %	79.98 %
Eff	26.59 %	27.04 %

- Spectral Mismatch factor SMM_{top}=1.0070; SMM_{bot}=0.9829.
- Designated illumination area defined by a thin metal mask was measured by a measuring microscope.
- Test results listed in this measurement report refer exclusively to the mentioned test sample.
- The results apply only at the time of the test, and do not imply future performance.

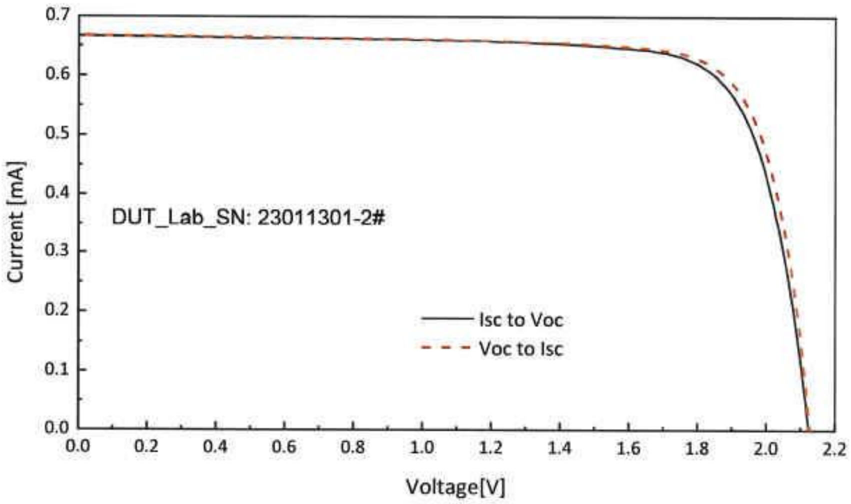

Fig.1 I-V curves of the measured sample

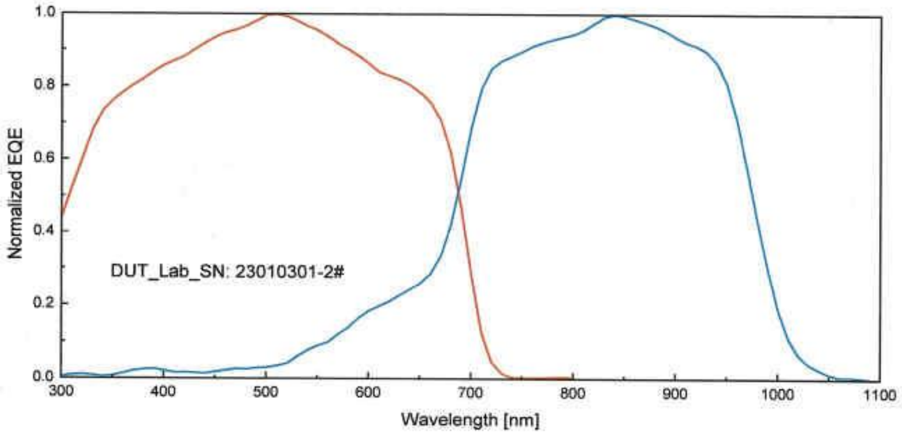

Fig.2 Normalized EQE curve of the measured sample

-------End of Report-------

Figure S11. Certification of photovoltaic performance of the tandem perovskite device measured by SIMIT. The FEDA tandem device has an independently certified PCE of 26.59% (27.04%) under reverse (forward) scan.

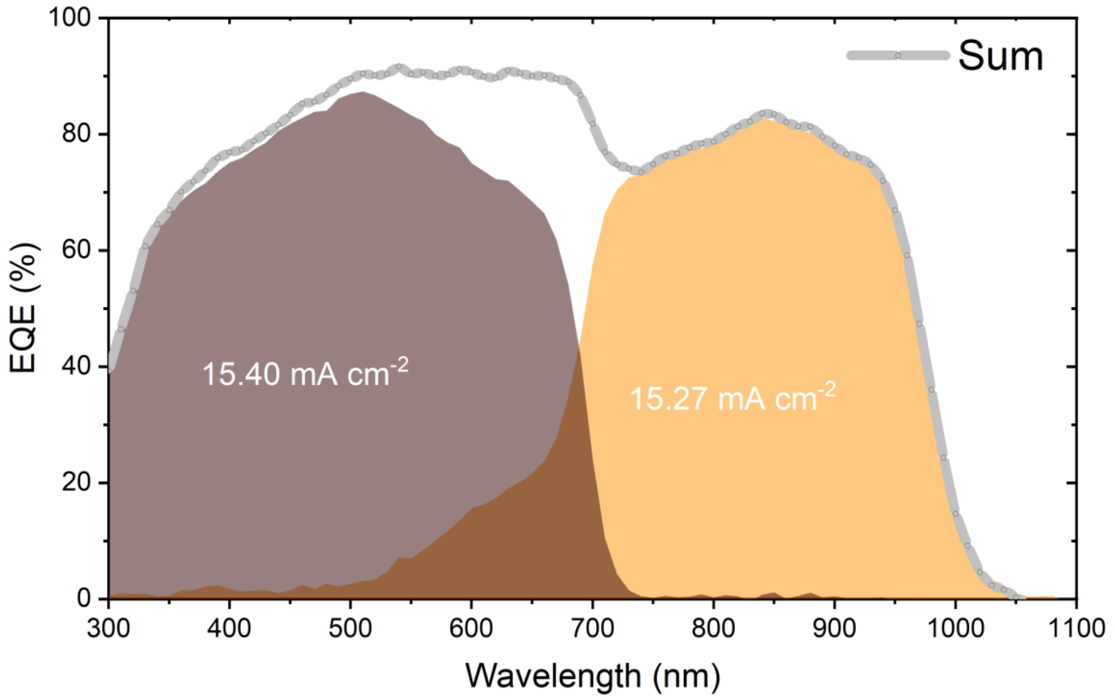

Figure S12. EQE spectra of tandem solar cells and the integrated J_{SC} values are 15.40 mA cm^{-2} and 15.27 mA cm^{-2} for top and bottom subcells, respectively.

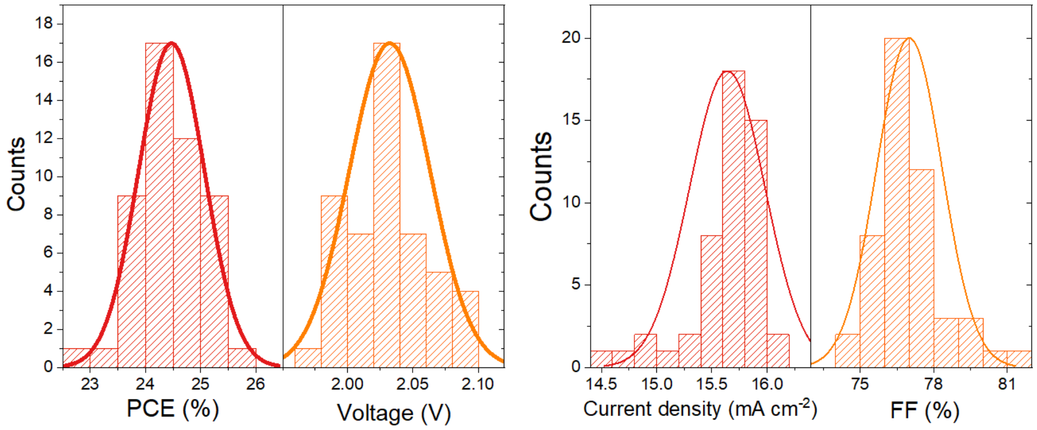

Figure S13. The statistic of FEDA film based tandem solar cells.

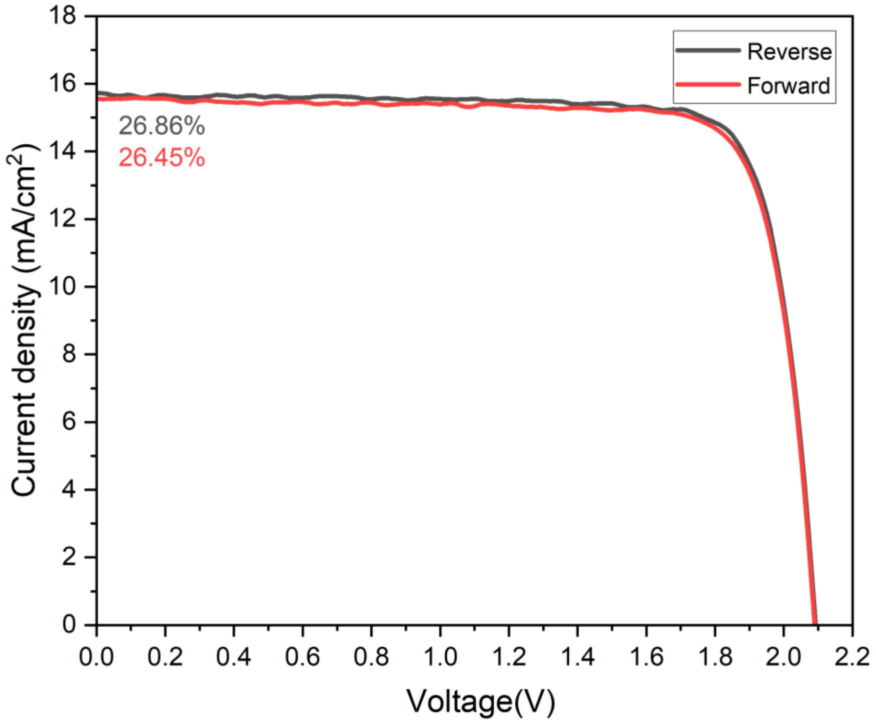

Figure S14 *J-V* curve of the best-performing TSC device (0.113 cm^2)

$$\text{Hysteresis Factors} = \frac{\text{PCE(reverse)} - \text{PCE(forward)}}{\text{PCE(reverse)}}$$

For our TSC device, the hysteresis factor of 0.042 cm^2 device is 0.016, and the hysteresis factor of 0.113 cm^2 is 0.015.

Table S1. Representative works about wide-bandgap (1.77 eV) solar cells. Parameters of the certified cell are shown in brackets.

Wide-bandgap perovskite (WBP)	E_{gWB}(eV)	PCE$_{WBP}$(%)	$V_{OC\text{-}WBP}$(V)	Ref
$FA_{0.8}Cs_{0.2}Pb(I_{0.62}Br_{0.38})_3$	1.76	17.3	1.22	Tan [9]
$FA_{0.8}Cs_{0.2}Pb(I_{0.6}Br_{0.4})_3$	1.77	16.7	1.23	Tan[10]
$FA_{0.8}Cs_{0.2}Pb(I_{0.6}Br_{0.4})_3$	1.77	16.5	1.216	Tan[11]
$FA_{0.8}Cs_{0.2}Pb(I_{0.6}Br_{0.4})_3$	1.77	15.9	1.206	Tan[12]
$Cs_{0.4}FA_{0.6}PbI_{1.95}Br_{1.05}$	1.78	16.0	1.23	Huang[13]
$FA_{0.6}Cs_{0.4}Pb(I_{0.65}Br_{0.35})_3$	1.80	16.3	1.22	Huang[14]
$FA_{0.8}Cs_{0.2}Pb(I_{0.7}Br_{0.3})_3$	1.75	14.0	1.143	Yan[15]
$Cs_{0.3}FA_{0.7}PbI_{2.1}Br_{0.9}$	1.75	17.1	1.225	Zhu[16]
$MA_{0.9}Cs_{0.1}Pb(I_{0.6}Br_{0.4})_3$	1.82	12.5	1.20	Jen[17]
$FA_{0.8}Cs_{0.2}Pb(I_{0.7}Br_{0.3})_3$	1.75	17.02	1.212	Li[18]
$FA_{0.8}Cs_{0.2}Pb(I_{0.6}Br_{0.4})_3$	1.79	20.2<br>**(19.3)**	1.33	Sargent[19]
$Cs_{0.3}FA_{0.6}DMA_{0.1}Pb(I_{0.7}Br_{0.3})_3$	1.75	20.3	1.33	Zhu[20]
$FA_{0.8}Cs_{0.2}Pb(I_{0.6}Br_{0.4})_3$	1.77	20.37<br>**(19.31)**	1.34	This work

Table S2. The data of the best narrow-bandgap, wide-bandgap, and all-perovskite tandem solar cells.

Device	PCE (%)	V_{OC} (V)	J_{SC} (mA cm^{-2})	FF (%)
WBP (Forward)	20.06	1.34	18.26	81.88
WBP (Reverse)	20.37	1.34	18.16	83.62
NBP (Forward)	21.39	0.84	32.06	79.02
NBP (Reverse)	20.48	0.84	32.24	75.77
Tandem(Forward)	27.23	2.12	16.01	80.36
Tandem(Reverse)	26.67	2.11	16.02	79.00

Reference

[1] Liao Y, Liu H, Zhou W, *et al*. Highly oriented low-dimensional tin halide perovskites with enhanced stability and photovoltaic performance. *J. Am. Chem. Soc* 2017; **139**: 6693-6699.

[2] Wang F, Jiang XY, Chen H, *et al*. 2d-quasi-2d-3d hierarchy structure for tin perovskite solar cells with enhanced efficiency and stability. *Joule* 2018; **2**: 2732-2743.

[3] Jiang Z. Gixsgui: A matlab toolbox for grazing-incidence x-ray scattering data visualization and reduction, and indexing of buried three-dimensional periodic nanostructured films. *J Appl Crystallogr* 2015; **48**: 917-926.

[4] Kresse G, Furthmuller J. Efficient iterative schemes for ab initio total-energy calculations using a plane-wave basis set. *Phys Rev B Condens Matter* 1996; **54**: 11169-11186.

[5] Kresse G, Furthmuller J. Efficiency of ab-initio total energy calculations for metals and semiconductors using a plane-wave basis set. *Comp Mater Sci*, 1996, **6**: 15-50.

[6] Blochl PE. Projector augmented-wave method. *Phys. Rev. B* 1994; **50**: 17953-17979.

[7] Perdew JP, Burke K, Ernzerhof M. Generalized gradient approximation made simple. *Phy Rev L* 1996; **77**: 3865-3868.

[8] Wang S, Sakurai T, Wen W, Qi Y. Energy level alignment at interfaces in metal halide perovskite solar cells. *Adv. Mater. Interfaces* 2018; **5**: 1800260.

[9] Lin R, Xu J, Wei M, *et al*. All-perovskite tandem solar cells with improved grain surface passivation. *Nature* 2022; **603**: 73-78.

[10] Wang Y, Gu S, Liu G, *et al*. Cross-linked hole transport layers for high-efficiency perovskite tandem solar cells. *Sci China Chem* 2021; **64**: 2025-2034.

[11] Lin R, Xiao K, Qin Z, *et al*. Monolithic all-perovskite tandem solar cells with 24.8% efficiency exploiting comproportionation to suppress sn(ii) oxidation in precursor ink. *Nat. Energy* 2019; **4**: 864-873.

[12] Xiao K, Lin R, Han Q, *et al*. All-perovskite tandem solar cells with 24.2% certified efficiency and area over 1 cm^2 using surface-anchoring zwitterionic antioxidant. *Nat. Energy* 2020; **5**: 870-880.

[13] Yu Z, Yang Z, Ni Z, *et al*. Simplified interconnection structure based on $C_{60}/SnO_{(2-x)}$for all-perovskite tandem solar cells. *Nat. Energy* 2020; **5**: 657-665.

[14] Yang Z, Yu Z, Wei H, *et al*. Enhancing electron diffusion length in narrow-bandgap perovskites

for efficient monolithic perovskite tandem solar cells. *Nat. Communications* 2019; **10**: 4498.
[15] Zhao D, Chen C, Wang C, *et al*. Efficient two-terminal all-perovskite tandem solar cells enabled by high-quality low-bandgap absorber layers. *Nat. Energy* 2018; **3**: 1093-1100.
[16] Tong J, Jiang Q, Ferguson AJ, *et al*. Carrier control in sn–pb perovskites via 2d cation engineering for all-perovskite tandem solar cells with improved efficiency and stability. *Nat. Energy* 2022; **7**: 642-651.
[17] Rajagopal A, Yang Z, Jo SB, *et al*. Highly efficient perovskite–perovskite tandem solar cells reaching 80% of the theoretical limit in photovoltage. *Adv. Mater*. 2017; **29**: 1702140.
[18] Wang C, Zhao Y, Ma T, *et al*. A universal close-space annealing strategy towards high-quality perovskite absorbers enabling efficient all-perovskite tandem solar cells. *Nat. Energy* 2022; **7**: 744-753.
[19] Chen H, Maxwell A, Li C, *et al*. Regulating surface potential maximizes voltage in all-perovskite tandems. *Nature* 2023; **613**: 676-681.
[20] Jiang Q, Tong J, Scheidt RA, *et al*. Compositional texture engineering for highly stable wide-bandgap perovskite solar cells. *Science* 2022; **378**: 1295-1300.
